# Association of *NAT2* genetic polymorphism with the efficacy of Neurotropin® for the enhancement of aggrecan gene expression in nucleus pulposus cells: a pilot study

**DOI:** 10.1186/s12920-021-00926-x

**Published:** 2021-03-11

**Authors:** Tomoko Nakai, Daisuke Sakai, Yoshihiko Nakamura, Natsumi Horikita, Erika Matsushita, Mitsuru Naiki, Masahiko Watanabe

**Affiliations:** 1 Department of Orthopaedic Surgery, Surgical Science, Tokai University School of Medicine, 143 Shimokasuya, Isehara, Kanagawa 259-1193 Japan; 2 Research Center for Regenerative Medicine and Cancer Stem Cell, Tokai University School of Medicine, 143 Shimokasuya, Isehara, Kanagawa 259-1193 Japan; 3 Institute of Bio-Active Science, Nippon Zoki Pharmaceutical Co., Ltd., Kinashi, Kato-shi, Hyogo 673-1461 Japan

**Keywords:** Polymorphism, *NAT2*, Rapid/intermediate acetylator, Nucleus pulposus cells, Intervertebral discs, Aggrecan, Neurotropin®

## Abstract

**Background:**

Intervertebral disc degeneration, one of the major causes of low-back pain, results from altered biosynthesis/turnover of extracellular matrix in the disc. Previously, we reported that the analgesic drug Neurotropin® (NTP) had an anabolic effect on glycosaminoglycan synthesis in cultured nucleus pulposus (NP) cells via the stimulation of chondroitin sulfate *N*-acetylgalactosaminyltransferase 1. However, its effect on the aggrecan core protein was not significantly detected, because of the data variance. A microarray analysis suggested that the effect of NTP on aggrecan was correlated with *N*-acetyltransferase 2 (NAT2), a drug-metabolizing enzyme. Specific *NAT2* alleles are known to correlate with rapid, intermediate, and slow acetylation activities and side effects of various drugs. We investigated the association between the efficacy of NTP on aggrecan expression and the *NAT2* genotype in cell donors.

**Methods:**

NP cells were isolated from intervertebral disc tissues donated by 31 Japanese patients (28–68 years) who underwent discectomy. NTP was added to the primary cell cultures and its effect on the aggrecan mRNA was analyzed using real-time quantitative PCR. To assess acetylator status, genotyping was performed based on the inferred *NAT2* haplotypes of five common single-nucleotide polymorphisms using allele-specific PCR.

**Results:**

The phenotype frequencies of *NAT2* in the patients were 0%, 42.0%, and 58.0% for slow, intermediate, and rapid acetylators, respectively. The proportions of responders to NTP treatment (aggrecan upregulation, ≥ 1.1-fold) in the intermediate and rapid acetylators were 76.9% and 38.9%, respectively. The odds ratio of the comparison of the intermediate acetylator status between responders and nonresponders was 5.2 (95% CI 1.06–26.0, P = 0.036), and regarding the 19 male patients, this was 14.0 (95% CI 1.54–127.2, *P* = 0.012). In the 12 females, the effect was not correlated with *NAT2* phenotype but seemed to become weaker along with aging.

**Conclusions:**

An intermediate acetylator status significantly favored the efficacy of NTP treatment to enhance aggrecan production in NP cells. In males, this tendency was detected with higher significance. This study provides suggestive data of the association between *NAT2* variants and the efficacy of NTP treatment. Given the small sample size, results should be further confirmed.

## Background

Intervertebral disc (IVD) degeneration is a chronic and progressive disease. Research on effective medical treatments for this disorder is ongoing. The IVD is a composite of substructures that consists of confining end plates on the superior and inferior faces, the highly fibrous anulus fibrosus on the outer periphery, and the highly hydrated nucleus pulposus (NP) at the center [1]. The IVD appears to be designed to sustain compression loads that are beneficial to it, as loading is the physiological stimulus for matrix turnover and induces matrix synthesis [2–4]. However, excessive loading can lead to deleterious changes in the IVD by downregulating the genes encoding anabolic proteins, with significant effects on aggrecan formation, while upregulating genes encoding matrix metalloproteinase [4], thereby inducing matrix degradation [5]. Aggrecan is the major noncollagenous component of the IVD. It is a large proteoglycan possessing numerous glycosaminoglycan (GAG) chains and a core protein, and is an integral part of the extracellular matrix in cartilaginous tissues. Its abundance and unique molecular features, i.e., the formation of aggregates in association with hyaluronan, provide the disc with its osmotic properties and ability to withstand compressive loads. The degradation and loss of aggrecan result in impairment of disc function and the onset of disc degeneration [6].

Previously, we reported that the analgesic drug Neurotropin® (NTP) had an anabolic effect on GAG synthesis in NP cells from the IVD via the stimulation of chondroitin sulfate *N*-acetylgalactosaminyltransferase 1 (CSGALNACT1), which initiates the synthesis of chondroitin sulfate (CS) polysaccharide chains attached to the core protein of aggrecan [7]. However, in our previous study, NTP did not significantly promote the expression of the gene encoding the aggrecan core protein, as it was decreased by the addition of NTP to the cells of a couple of donors. If we anticipate a new application of NTP as a medicine for the restoration of deteriorated disc matrix, NTP should also increase or at least maintain the expression level of the aggrecan core protein, to anchor the increased CS side chains onto the cell surface in association with hyaluronan. To explain the large variance in our previous data, in the current study, we investigated whether the difference in cellular responsiveness to NTP stems from the genetic background of the donors.

NTP, a nonprotein extract of inflamed rabbit skin inoculated with the vaccinia virus, has been used in Japan to treat chronic pain via oral, intramuscular, or intravenous administration [8], and was reported to provide effective relief for various types of pain, such as headache, low-back pain, neck–shoulder–arm syndrome, postherpetic neuralgia, and fibromyalgia [8–11]. Despite its clinical advantages, the characteristics of NTP remain unclear regarding two issues: first, its main active ingredient is unclear because NTP comprises many components, including nucleic acids, amino acids, and sugars [12]; second, the mechanism underlying the local action of this reagent is not clearly understood, although the main effect of NTP has been reported to be the activation of the descending monoaminergic pain inhibitory systems of the central pain pathway [13].

**Fig. 1:**
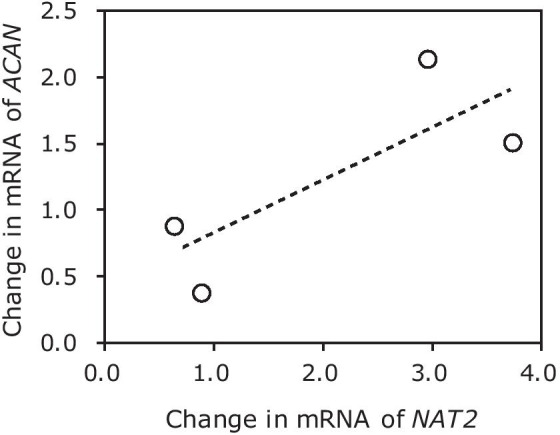
Correlation between the expression of the aggrecan (*ACAN*) and *N*-acetyltransferase 2 (*NAT2*) genes induced by NTP. The fold changes in mRNA expression induced by NTP treatment in cultured NP cells are shown (N = 4). *ACAN* and *NAT2* were detected by qPCR and microarray analysis (data available on the NCBI repository), respectively

To identify the genetic basis of the large variance in our previous study we re-explored the microarray data generated previously to investigate comprehensively the gene expression changes in NTP-treated NP cells from four patients (all data are available on the Gene Expression Omnibus repository, https://www.ncbi.nlm.nih.gov/gds/?term=GSE114169). The gene encoding arylamine *N*-acetyltransferase 2 (NAT2) appeared to be correlated with cell donor responsiveness to NTP regarding aggrecan gene expression (Fig. 1). NAT2, a drug-metabolizing enzyme, is one of two structurally related isoenzymes, NAT1 and NAT2. These NATs are phase II xenobiotic metabolism enzymes that catalyze the detoxification of arylamines via *N*-acetylation and the bioactivation of *N*-arylhydroxylamines by *O*-acetylation. NAT2 acetylates a large variety of arylamine-acceptor structures, such as caffeine, procainamide, and sulfasalazine, as well as the antituberculosis drug isoniazid [14–16]. Specific types of *NAT2* alleles are known to be correlated with distinct metabolic activities; patients with a NAT2 that is inactive against isoniazid have been reported to have a higher risk of developing antituberculosis-drug-induced liver injury [16–20]. Genotypic polymorphisms at the *NAT2* locus give rise to either the “slow” or the “rapid” acetylator phenotype, as well as the “intermediate” acetylator phenotype in “slow/rapid” heterozygotes [21]. These phenotypes also affect individual variation in cancer susceptibility, responses to environmental toxins, and the effectiveness of prescribed medications [22, 23].

We therefore hypothesized that genetic polymorphism in *NAT2* affects the modulation of the expression of the aggrecan mRNA by NTP treatment, because NTP possibly includes components with arylamine-acceptor structures. Our objective was to investigate the association between the promoting effect of NTP on aggrecan gene expression in NP cells and the *NAT2* genotype status in the cell donors.

## Methods

### Study population

We conducted a cross-sectional study in the cultured cells donated from 31 Japanese patients (aged 28–68 years) who underwent discectomy at Tokai University Hospital from January 2019 to March 2020. The surgical-waste samples consisted of lumbar IVDs from herniation, and vertebral and spinal fusions with two bursts from thoracic and lumbar IVDs. The frequency of females was 38.7%, and the mean age ± standard deviation (SD) of the female and male donors was 52.0 ± 12.2 and 52.5 ± 11.9 years, respectively.

### Tissue culture and cell isolation and expansion

The NP tissue was carefully separated from the anulus fibrosus. We used NP tissue for our experiments when at least 0.05 g of wet weight was obtained. The sample was cut into small pieces and incubated in α-minimal essential medium (α-MEM; FUJIFILM Wako Pure Chemical, Osaka, Japan) supplemented with 10% fetal bovine serum (FBS; Sigma-Aldrich, St. Louis, MO), 100 U/ml penicillin (Thermo Fisher Scientific, Waltham, MA), and 100 mg/ml streptomycin (Thermo Fisher Scientific) at 37 °C and 5% CO_2_ for 2 weeks. The cultured NP tissues were collected and digested with TrypLE Express (Thermo Fisher Scientific) for 30 min, followed by incubation with 0.25 mg/ml Collagenase-P (Roche, Basel, Switzerland) for 80 min at 37 °C. The isolated cells were washed twice with α-MEM and seeded at a density of approximately 5 × 10^3^ cells/cm^2^. Cells were cultured in α-MEM supplemented with 10% FBS, 100 U/ml penicillin, and 100 mg/ml streptomycin at 37 °C and 5% CO_2_ under hypoxic conditions of 2% O_2_. The medium was replaced twice a week and the cells were trypsinized (Thermo Fisher Scientific) and subcultured before they reached confluence. Cells harvested from second-passage cultures were used for experiments with NTP; thereafter, further-passaged cells were used to extract genomic DNA.

### Chemicals

NTP was provided by Nippon Zoki Pharmaceutical Co., Ltd. (Osaka, Japan). The biological activity of NTP was expressed in NTP Units (NU). l-ascorbic acid 2-phosphate (AsAP) was purchased from FUJIFILM Wako Pure Chemical.

### Treatment of cultured NP cells with NTP

NP cells were seeded in 6-well culture plates at a density of 5 × 10^3^ cells/cm^2^ on “day zero” and were cultured in α-MEM containing 10% FBS (basal medium) overnight prior to NTP addition. As reported previously, the cells were stimulated with NTP dissolved in fresh α-MEM supplemented with 10% FBS and 50 μg/ml AsAP. The medium was replaced every second day for 1 week. The NTP concentrations were set at 0.1 and 0.2 mNU/ml. The concentration of NTP in the culture media was set to its approximate levels in the blood plasma when taken according to the clinical prescriptions. On days 6 and 8, NP cells were harvested and the expression of the aggrecan gene was evaluated.

### Real-time quantitative PCR

Cells cultured in the presence or absence of NTP treatment were harvested and homogenized in lysis buffer, and total RNA was prepared using an SV Total RNA Isolation System (Promega, Madison, WI). For each sample, 2 μg of total RNA was reverse transcribed into cDNA using a High Capacity RNA-to-cDNA kit (Life Technologies, Waltham, MA). Relative quantification of the target mRNA was performed using the comparative C_T_ method with the sets of primers and probes for the endogenous control glyceraldehyde-3-phosphate dehydrogenase gene (*GAPDH*, Hs99999905_m1), and the target genes of aggrecan *(ACAN)* (Hs00153936_m1) and *CSGALNACT1* (Hs00218054_m1), all of which were provided as predeveloped TaqMan Gene Expression Assay Reagents (Life Technologies). The assay for *ACAN* covers the mRNAs of transcript variants for the aggrecan core protein, including variants of 1, 2, X2, and X3. The PCR amplification and analysis were performed on a QuantStudio 3 real-time PCR instrument (Life Technologies).

NTP was considered effective when the expression of the *ACAN* mRNA was increased by more than 1.1-fold compared with the control culture in the basal medium. For each donor, the highest values obtained in four experimental conditions (treated with two dose settings and harvested at two time points) were selected and analyzed.

### *NAT2* genotyping and assignment of acetylator phenotypes

Subcultured NP cells were lysed and genomic DNA was purified using a DNA purification kit (NucleoSpin® Tissue; Macherey–Nagel GmbH, Düren, Germany). DNA was quantified using a NANODROP LITE spectrophotometer (Thermo Fisher Scientific) and was stored at –30 °C until use. Five single-nucleotide polymorphisms (SNPs) were analyzed: *rs*1041983 (282C > T), *rs*1801280 (341 T > C), *rs*1799929 (481C > T), *rs*1799930 (590 G > A), and *rs*1799931 (857 G > A). The genotyping assays using PCR were performed based on a fundamental procedure [24] and the manuals from the reagent provider (Life Technologies). Briefly, a 20 × primer and probe mix from TaqMan Drug Metabolism Assays (Life Technologies) were supplied for each assay, and a 1 × concentration of this mix, as well as a half volume of 2 × TaqPath ProAmp Master Mix (Life Technologies) and 10 ng of the genomic DNA sample, were added to each well. The thermal conditions of the experiment were 95 °C for 5 min, followed by 45 cycles of 95 °C for 15 s and 60 °C for 1 min. The PCR amplification and endpoint reading were performed on a QuantStudio 3 real-time PCR instrument. In each experiment, control samples with no DNA template were run to ensure that there was no amplification of contaminating DNA. Cell donors possessing rapid/rapid homozygous *NAT2* alleles were classified as rapid acetylators, individuals possessing a rapid/slow heterozygous genotype were classified as intermediate acetylators, and individuals possessing a slow/slow homozygous genotype were classified as slow acetylators. The inference of haplotype–phenotype information pertaining to the NAT2 acetylator status was determined based on the annotation table provided in Human NAT2 Alleles (Haplotypes), a database built by Democritus University of Thrace (http://nat.mbg.duth.gr/Human%20NAT2%20alleles_2013.htm).

### Statistical analysis

The calculations of odds ratios (ORs) and Pearson’s chi-squared (χ^2^) test (to obtain *P*-values) were performed in 2 × 2 tables using the Statistical Analysis WEB-BellCurve software (https://bellcurve.jp/statistics). The calculation of 95% confidence intervals (95% CIs) was carried out using the Microsoft Excel 2016 software according to the equation:

$${\text{ln }}\left( {95{\text{\% }}\;{\text{ CI}}} \right) = {\text{ln }}\left( {{\text{OR}}} \right) \pm 1.96\sqrt {1/{\text{a}} + 1/{\text{b}} + 1/{\text{c}} + 1/{\text{d}}} ,$$

where a is the number of responders with variant *NAT2* haplotypes, b is the number of nonresponders with variant *NAT2* haplotypes, c is the number of responders with the wild-type homozygous *NAT2* genotype, d is the number of nonresponders with the wild-type homozygous *NAT2* genotype, and “ln” stands for natural logarithm. In addition, the following analyses were performed using the functions of the Microsoft Excel 2016 software: Student’s *t*-test and F-test were applied to compare the differences between two groups regarding phenotype and changes in the levels of the aggrecan mRNA; the correlation coefficient between changes in the aggrecan mRNA after NTP treatment and donor’s age was calculated; and Student’s *t*-distribution test for its significance was performed. Significance was set at *P* < 0.05.

### Compliance with ethical standards

This study, including the use of patient-derived surgical-waste material and sequence analysis of genomic DNA, was approved by the Clinical Research Ethics Committee of Tokai University School of Medicine (study code: 18I-25), and was conducted in accordance with approved protocols. Informed consent forms with written provision were completed by all patients before the donation of IVD samples.

## Results

We identified four haplotypes, NAT2*4, NAT2*5B, NAT2*6A, and NAT2*7B, in the 31 donors (Table 1). The wild-type NAT2*4 haplotype was present in 79.0% of the examined subjects, whereas the remaining three haplotypes were variants. There was no significant difference (Pearson χ^2^ test) in the distributions of haplotypes between our data and the data from a study that assessed 200 healthy Japanese volunteers [25]. As exceptions, the NAT2*11 and NAT2*13 haplotypes, which are present in 0.25% and 1.25% of the Japanese population, respectively, were not detected in the cell donors enrolled in our study.

**Table 1:** Distribution of *NAT2* haplotypes in the cell donors and the Japanese population

Allele	Nucleotide change(s)	Amino acid change(s)	Type	Freq. (%) detected	Freq. (%) literature^a^	*P* value ^b^
NAT2*4	–	–	Rapid	79.0	69.5	0.125
NAT2*5B	T341C, C481T, A803G	Ile114Thr, Lys268Arg	Slow	1.6	0.5	0.317
NAT2*6A	C282T, G590A	Arg197Gln	Slow	9.7	19.8	0.056
NAT2*7B	C282T, G857A	Gly286Glu	Slow	9.7	8.8	0.811

^a^Allele frequency in the literature, the data was from healthy 200 volunteers ranged 20–60 years old

^b^Calculated for haplotype frequencies

Next, genotyping was performed to assess the acetylator status based on the inferred *NAT2* haplotypes. We identified four genotypes, NAT2*4/*4, NAT2*4/*5B, NAT2*4/*6A, and NAT2*4/*7B, at frequencies of 58.0%, 3.2%, 19.4%, and 19.4%, respectively (Table 2 Panel A). The NAT2*4/*4 alleles of rapid/rapid homozygotes were classified as a rapid acetylator phenotype, whereas rapid/slow heterozygotes, i.e., with NAT2*4/*5B, NAT2*4/*6A, and NAT2*4/*7B alleles, were classified as an intermediate acetylator phenotype. However, none of the individuals were slow/slow homozygotes, i.e., a slow acetylator phenotype. The results of the statistical analysis showed that no single genotype was positively correlated with the efficacy of NTP treatment (Pearson χ^2^ test). Subsequently, we analyzed the differences between phenotypes regarding the response to NTP treatment. The phenotype frequencies of *NAT2* in the donors were 58.0% and 42.0% for rapid (NAT2*4/*4) and intermediate (NAT2*4/*5B, NAT2*4/*6A, and NAT2*4/*7B combined) acetylators, respectively.

**Table 2:** *NAT2* genotype frequencies and response to NTP treatment

Genotype	N	% ^b^	Phenotype	NTP+ N^c^	NTP+ %^d^	OR (95%CI)	*P* value
Panel A							
*4/*4	18	58.0	Rap	7	38.9	0.19 (0.04–0.95)	0.036
*4/*5B	1	3.2	Int	1	100	–	–
*4/*6A	6	19.4	Int	4	66.7	1.9 (0.28–11.98)	0.517
*4/*7B	6	19.4	Int	5	83.3	5.4 (0.55–53.27)	0.118
*4/*5B,*6A,*7B ^a^	13	42.0	Int	10	76.9	5.2 (1.06–26.0)	0.036

Genotype in genders	N	%^b^	Phenotype	NTP+ N^c^	NTP+ %^d^	OR (95%CI)	*P* value
Panel B							
Male *4/*4	9	29.0	Rap	2	22.2	0.1 (0.01–0.65)	0.012
*4/*5B,*6A,*7B^a^	10	32.3	Int	8	80.0	14.0 (1.54–127.2)	0.012
Female *4/*4	9	29.0	Rap	5	55.5	0.6 (0.04–0.97)	0.735
*4/*5B,*6A,*7B^a^	3	9.7	Int	2	66.7	1.6 (0.10–24.7)	0.735

*Rap.* Rapid, *Int.* intermediate

^a^Total of the variants

^b^Frequency in the total sample

^c^Responders to NTP treatment

^d^Frequency of responders in each group

Regarding with the efficacy of NTP, 38.9% and 76.9% of the individuals with rapid and intermediate acetylator phenotypes, respectively, were responders to NTP treatment (aggrecan upregulation, ≥ 1.1-fold). The OR of the comparison of the intermediate acetylator phenotype between responders and nonresponders was 5.2 (95% CI 1.06–26.0, *P* = 0.036, Pearson χ^2^ test), which suggests that the intermediate acetylator phenotype was significantly correlated with the efficacy of the NTP treatment. The gender-specific statistics show that the frequency of responders among male and female intermediate acetylator individuals was 80.0% and 66.7%, respectively (Table 2 Panel B). In male donors, the OR of the comparison of the intermediate acetylator phenotype between responders and nonresponders was 14.0 (95% CI 1.54–127.2, *P* = 0.012, Pearson χ^2^ test). In contrast, in female donors, the frequency of responders was not significantly correlated with the *NAT2* phenotype.

### Phenotype and age-related changes in the expression of the aggrecan mRNA after NTP treatment

**Fig. 2:**
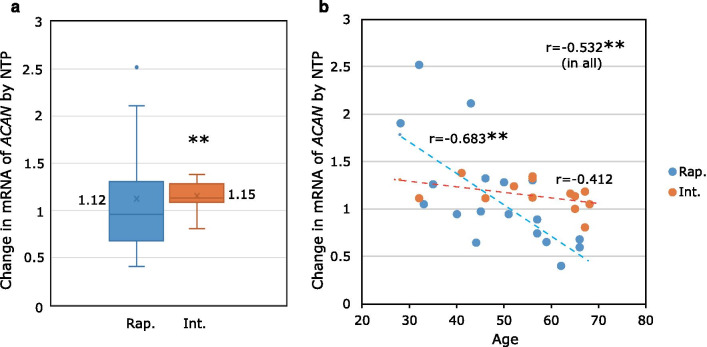
Phenotype and age-related changes in the mRNA expression of *ACAN* induced by NTP treatment. The fold changes in the mRNA expression of *ACAN* induced by NTP treatment compared with the control in cultured NP cells are shown (N = 31). **a** Comparison between *NAT2* phenotypes. Blue denotes the rapid acetylator phenotype (Rap., N = 18) and orange denotes the intermediate acetylator phenotype (Int., N = 13). There was a significant increase compared with the control in the intermediate acetylator phenotype (*P* = 0.002). **b** The data from **a** are plotted against the age (years) of the donors. There was a weakly negative correlation with age in the rapid acetylator phenotype (*P* = 0.002) and in all data (*P* = 0.0021). The blue and orange symbols are as described in **a**

The quantitative data pertaining to the changes in the expression levels of the aggrecan mRNA after NTP treatment compared with each control were plotted against the *NAT2* phenotypes (Fig. 2a). As shown in the box-whisker plots, there was a large variance in the rapid acetylator phenotype; consequently, no significant effect of the NTP treatment was detected compared with the control (mean ± SD, 1.12 ± 0.56, *P* = 0.187, Student’s *t*-test), while there was a significant increase in the intermediate acetylator phenotype (mean ± SD, 1.15 ± 0.16, *P* = 0.002, Student’s *t*-test). Although the mean values of each phenotype were similar (1.12 and 1.15, respectively), there was a statistically significant difference in the distributions of data between the rapid and intermediate acetylator phenotypes (*P* = 2.69E−05, F-test). The change in the expression of the aggrecan mRNA after NTP administration is plotted against donor’s age in Fig. 2b. There was a weakly negative correlation with age in all data (*r* = − 0.532, *P* = 0.0021, Student’s *t*-distribution test). Regarding the rapid acetylator phenotype, the same tendency was found at higher significance (*r* = − 0.683, *P* = 0.002, Student’s *t*-distribution test). In contrast, no significant correlation was found for the intermediate acetylator phenotype.

#### Male donors classified as having an intermediate acetylator phenotype exhibited a favorable response to NTP treatment

**Fig. 3:**
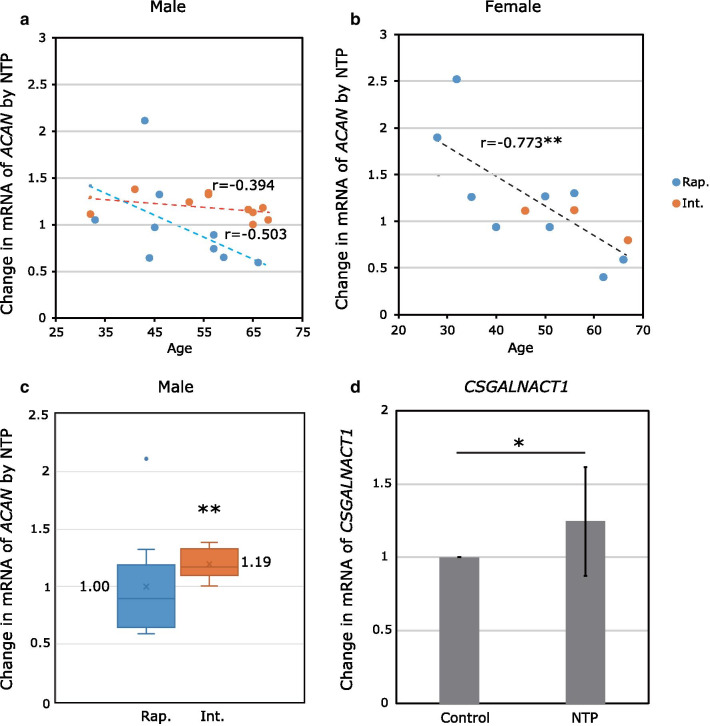
Gender-specific analysis of the changes in the mRNA expression of *ACAN* and reconfirmation of *CSGALNACT1*. The fold changes in the mRNA expression of *ACAN* induced by NTP treatment in cultured NP cells are shown. Blue denotes the rapid acetylator phenotype (Rap.) and orange denotes the intermediate acetylator phenotype (Int.). **a**, **b** The data from the male and female donors are plotted against age (years), respectively (Male Rap.: N = 9, Int.: N = 10; Female Rap.: N = 9, Int.: N = 3). A significantly negative correlation with age was observed in the female donors (*P* = 0.006). **c** Comparison between *NAT2* phenotypes in the male donors. Mean values are indicated. There was a significant increase compared with the control in the intermediate acetylator phenotype (*P* = 0.001). **d** Changes in the mRNA expression of *CSGALNACT1* induced by NTP treatment compared with the control. The cultured NP cells from impartially selected donors were used as experimental samples (N = 10, including rapid:intermediate = 5:5, responder:nonresponder = 5:5, and female:male = 4:6). NTP treatment significantly increased the expression of the *CSGALNACT1* mRNA in NP cells compared with the control (*P* = 0.013)

Gender-specific analyses are shown in Fig. 3a, b. An age-related correlation was not observed in the male donors, while a significantly negative correlation was observed in the female donors (*r* = − 0.773, N = 12, *P* = 0.006, Student’s *t*-distribution test). In Fig. 3c, data from the male donors are plotted according to *NAT2* phenotype. As shown in the box-whisker plots, there was a large variance in the rapid acetylator phenotype; consequently, no significant effect of the NTP treatment was detected compared with the control (mean ± SD, 1.00 ± 0.48, N = 9, *P* = 0.98, Student’s *t*-test), while there was a significant increase in the intermediate acetylator phenotype (mean ± SD, 1.19 ± 0.13, N = 10, *P* = 0.001, Student’s *t*-test). Moreover, there was a statistically significant difference in the distributions of the data between the rapid and intermediate acetylator phenotypes (*P* = 2.8E−04, F-test). These results suggest that male donors classified as having an intermediate acetylator phenotype are favorable responders to NTP treatment.

### Reconfirmation of efficacy of NTP on expression of the *CSGALNACT1* mRNA

To confirm the efficacy of NTP that we reported previously [7], the changes in the relative expression of the *CSGALNACT1* mRNA were examined (Fig. 3d). Ten samples were impartially selected according to the results of *NAT2* phenotype (rapid:intermediate = 5:5), responsiveness (responder:nonresponder = 5:5), and gender (female:male = 4:6) presented above. Quantitative PCR showed that NTP treatment significantly increased the expression of the *CSGALNACT1* mRNA in NP cells compared with the control (mean ± SD, 1.28 ± 0.37, N = 10, *P* = 0.013, Student’s *t*-test).

## Discussion

Although there is insufficient evidence to make a recommendation for or against an association between low back pain and lumbar degenerative changes, including intervertebral disc degeneration using imaging, level III evidence exists on the fact that presence of midline low back pain increases the probability of lumbar intervertebral disc degeneration[26, 27]. It is well documented in the literature that degeneration of the intervertebral disc accounts for the onset of low-back pain resulting from altered biosynthesis/turnover of extracellular matrix in the intervertebral disc[5, 6]. Because NTP had been used clinically to treat low back pain in Japan, we previously showed NTP’s anabolic effect on biosynthesis/turnover of extracellular matrix by intervertebral disc cells to define insights to possible mechanism of action [7].

In the current study, we showed that the NAT2 intermediate acetylator phenotype (comprising the NAT2*4/*5B, NAT2*4/*6A, and NAT2*4/*7B genotypes) was associated with the effectiveness of NTP regarding the promotion of the expression of the aggrecan mRNA in cultured NP cells. Thus, *NAT2* may be one of the genetic factors that act as a watershed that separates the presence or absence of negative effects of NTP in cultured NP cells. In contrast, we did not find any significant differences between intermediate and rapid (homozygous for the NAT2*4 allele) acetylator phenotypes regarding their mean values of upregulation of aggrecan mRNA expression (Fig. 2a). This was because a few strongly positive responses by the cells from young donors (< 45 years) counterbalanced the negative responses by the cells from older donors (> 45 years) in the rapid phenotype group (Fig. 2b). This age-related variance in cellular responsiveness was also found among the female donors (Fig. 3b).

A study of middle-aged and elderly postmenopausal women with exogenous estrogen therapy reported that neither estrogen concentration nor age was correlated with NAT2 activities, as measured by the caffeine metabolic ratio [28]. In another study that enrolled children of various ages, including infants, discordance between phenotype (acetylation) and genotype (*NAT2*) was reported [29]. In contrast, during the development of the outbred CD-1 mouse strain, a gender-dependent difference was observed; the kidney p-aminobenzoic acid/Nat2-acetylating activity of female mice showed a 2.5-fold increase at day 80 compared with day 1, whereas males showed a 4.3-fold increase at day 25 and a 5.8-fold increase at day 80 [30]. These findings provided knowledge about the difference between genders and the age-related changes in the function of NAT2, which currently exhibit diverse aspects; thus, it remains unclear whether any changes occur in age- or gender-specific manners.

Generally, *NAT2* genetic variants have been linked to decreased enzymatic activity and variable stability, leading to an imbalance in the xenobiotic detoxification and increased susceptibility to different forms of cancer [22, 31]. Nevertheless, the rapid *NAT2* phenotype has been reported to metabolically activate the toxicity of xenobiotic substances, such as *N*-hydroxylated heterocyclic aromatic amines (HAAs) via *O*-acetylation, to form the reactive *N*-acetoxy species. Some HAAs are formed when meat is cooked at high temperature for a long time, and high HAA intake has been associated with an increased risk of colorectal cancer compared with the intermediate/slow acetylator phenotypes [32]. Therefore, *NAT2* with a rapid phenotype seems to activate environmental toxins in some cases, in addition to catalyzing several pharmacologically and toxicologically significant detoxification reactions [33]. Moreover, a significant association between the NAT*6A polymorphism and age-related hearing loss has been reported: the genetic effect on presbycusis stemmed from the observation that NATs, together with cytochrome P450 and glutathione S-transferases, metabolize a wide range of xenobiotics and are important for the balance of oxidative status to protect cells against environmental toxins and the cellular damage caused by oxidative free radicals [34]. Therefore, the arylamine-catalyzing ability of NAT2 combined with other factors of the cultured NP cells may also be implicated in the current observations.

Regarding the functional mechanism underlying the effect of NTP on cultured NP cells, we previously reported that NTP activates the PI3–AKT pathway and promotes the synthesis of sulfated GAGs, such as chondroitin sulfate, heparin sulfate, and keratin sulfate. As one of the key effectors of the function of NTP, we demonstrated an increase in the levels of the CSGALNACT1 enzyme, which initiates the synthesis of CS polysaccharide chains [7]. In the current study, we detected a *NAT2* phenotype-dependent increase in the expression levels of the aggrecan mRNA induced by NTP in cultured NP cells. According to a previous report [6], aggrecan abundance reaches a plateau in the early twenties, declining thereafter because of proteolysis, mainly by matrix metalloproteinases and aggrecanases, although the degradation of hyaluronan and nonenzymatic glycation may also participate in this process. Aggrecan loss is an early event in disc degeneration, although it is a lengthy process.

In the current study, the male donors classified as having an intermediate acetylator phenotype exhibited the highest significance in the correlation with the frequency of responders to NTP (OR = 14.0; 95% CI 1.54–127.2; *P* = 0.012, Pearson χ^2^ test). It is noteworthy that no cells from individuals possessing an intermediate acetylator phenotype, including donors in their late sixties, were affected negatively by NTP (Fig. 3a, c). Regarding the age-related deterioration of human NP tissue, we previously reported an exhaustion of NP progenitor cells with evidence of an exponential decline in the frequency of Tie2-positive cells in freshly isolated cells from NP tissues donated by 23 patients (aged 19–70 years) [35]. Therefore, encouraging NP cells to produce aggrecan via NTP treatment will be beneficial for elderly male patients, even if the effect is *NAT2* phenotype-specific. As we have demonstrated that NTP treatment significantly increased the expression of the *CSGALNACT1* mRNA in the cells from 10 impartially selected individuals, it is likely that the promoting effect of NTP regarding the expression of CS side chains is also reliable.

Our study had several limitations. First, our sample size was small, especially the number of female individuals who possessed *NAT2* variants (N = 3), which precluded comparisons with those individuals with the rapid phenotype (N = 9). Therefore, the negative correlation between age and the effectiveness of NTP observed in females might be attributed to the tendency toward a *NAT2* rapid phenotype in this population (75% of females). As we investigated the cells derived from surgically removed IVD tissues, the composition of the donors was dependent on the morbidity of the disc diseases that required discectomy. The frequency of female patients in the current study (38.7%) was not largely different from the frequency of female patients reported in the literature (35.2–35.6%), among whom SNPs in the *THB2* and *SKT* genes were reported to be responsible for the susceptibility to disc diseases, such as lumbar disc herniation, in Japan [36, 37]. Moreover, the median age of the donors was 56 years. Therefore, it is difficult to recruit cell donors without bias in sex and age. Second, the main components of NTP that activate the PI3–AKT pathway have not been elucidated, and there are no clues regarding whether NTP contains arylamine or a large variety of its analogs. Further constituent analyses of NTP are hence needed to identify its promoting or inhibitory active ingredients, to develop a new purification technique to customize NTP for IVD regeneration through the upregulation of aggrecan synthesis in the disc.

As the current study is consisted with the small number of samples, we tried to make it more substantial by incorporating other publicly available dataset because recent advanced statistical methods may enable us to collect and analyze publicly available gene expression profiling dataset for identification of key genes as potential biomarkers responding to the drug efficacy. With a combined large size of samples, we might be able to construct co-expression network with weighted gene co-expression network R package [38], however, there were only three datasets of microarray and one RNA-sequence, none of which related to NTP. If we collected additional NTP-related datasets originated from human NP cells, we could have explored not only an inferred correlation patterns between two genes but also covered neighborhood across expression data through constructing subnetworks. Such a systematic approach might have led us to identify hub genes responsible for the NTP treatment along with protein–protein interactions network [39].

Also a meta-analysis is useful to increase statistical power of the studies having limitations due to the insufficient sample size. Briefly, using keywords related to the study, an initial screening of all titles and/or abstracts of published articles is performed and followed by careful full text check. As one of the validation criteria, relative risk estimates should have been reported with standard errors or 95% CIs. Data are then extracted from the validated articles [40, 41]. Dosage effect and age or gender specificity should be considered when evaluating the response to NTP treatment. In fact, we found only one article regarding effect of NTP on cultured NP cells from five men and one woman aged 23–31 years [13], however, reported target gene was not aggrecan, but *COX2, TNFα*, and *PLA2* were examined to discuss its anti-inflammatory effect. Unlike peripheral blood, chance of research-use of intervertebral disc tissue is scarce, therefore, the organ specific database needs to be reinforced as much as possible.

## Conclusions

Overall, our study suggests an association between the promoting effect of NTP on aggrecan gene expression in NP cells and genetic polymorphisms in *NAT2* in cell donors. The *NAT2* intermediate acetylator phenotype significantly favored the effectiveness of the NTP treatment by enhancing aggrecan production in NP cells. In particular, males classified as having this phenotype were the most feasible group. The above evidence suggests the potential value of *NAT2* genotyping in the selection of patients that respond to NTP treatment, which represents a new application of NTP as a medicine for the restoration of deteriorated disc matrix. Given the small size of samples, further studies including larger cohorts are needed.

## Data Availability

The data that support the findings of this study are available from the corresponding author D.S., upon reasonable requests. The individual genomic data are not publicly available due to privacy or ethical restrictions. The microarray data are available in the Gene Expression Omnibus repository, https://www.ncbi.nlm.nih.gov/gds/?term=GSE114169. The SNP data is available on a database built by Democritus University of Thrace, http://nat.mbg.duth.gr/Human%20NAT2%20alleles_2013.htm.

## Abbreviations

NTP: Neurotropin®
NAT2: *N*-Acetyltransferase 2
IVD: Intervertebral disc
NP: Nucleus pulposus
ACAN: Aggrecan
GAG: Glycosaminoglycan
CS: Chondroitin sulfate
CSGALNACT1: Chondroitin sulfate *N*-acetylgalactosaminyltransferase 1
SNP: Single-nucleotide polymorphism
OR: Odds ratio
CIs: Confidence intervals
HAAs: *N*-Hydroxylated heterocyclic aromatic amines
PI3–AKT: Phosphatidylinositol 3-kinase which activates protein kinase B
*THB2*: Thrombospondin 2
*SKT*: Sickle tail
